# Agentic LLM reasoning in pediatric diabetes outcome prediction from multimodal medical records

**Published:** 2026-10-29

**Authors:** Daniel Palacios, Guantong Qi, Anna Kadi, Natali Borrego, Sukru Aras, Michael L. Ferm, Elizabeth Kubota-Mishra, Maria J. Redondo, Zhandong Liu, Mustafa Tosur, Hu Chen

## Abstract

Large language model (LLM) agents are proposed for clinical prediction but are untested in pediatrics, where cohorts are small and outcomes are uncommon. Using records from 2,489 youth with type 2 diabetes (T2D) and 4,018 with type 1 diabetes (T1D), spanning structured data, note-derived social determinants and, in T1D, continuous glucose monitoring data, we predicted seven T2D outcomes, four also in T1D, at 24 months from 18 months of follow-up. CEDAR (Clinical Evidence-Driven Agent for Risk-prediction), an evidence-citing, self-auditing LLM pipeline, outperformed trained machine learning in both cohorts (pooled area under the receiver operating characteristic curve 0.815 versus 0.656 to 0.700 in T2D and 0.756 versus 0.603 to 0.631 in T1D), most on the sparsely screened outcomes. Only 4 of 70,246 values it cited did not match the chart. Reading only the evidence and reasoning cited by CEDAR, two pediatric endocrinologists reached the same prediction as CEDAR in about nine of ten cases, agreeing with each other on 90.5%.

